# Initial treatment approaches and healthcare utilization among veterans with low back pain: a propensity score analysis

**DOI:** 10.1186/s12913-023-09207-y

**Published:** 2023-03-21

**Authors:** Catherine Schmidt, Matthew Borgia, Tingting Zhang, Perman Gochyyev, Theresa I. Shireman, Linda Resnik

**Affiliations:** 1 Department of Physical Therapy, MGH Institute of Health Professions, 36 1st Avenue, Boston, MA 02129-4557 USA; 2 Providence VA Medical Center, 830 Chalkstone Avenue, Providence, RI 02912 USA; 3 Department of Health Services, Policy and Practice: Center for Gerontology and Health Care Research, Brown University, Providence, RI 02912 USA

**Keywords:** Physical therapy, Rehabilitation, Low back pain, Physiotherapy, Health care utilization, Spinal surgery, Opioids, Spinal pain, Propensity score analysis

## Abstract

**Background:**

Opioid prescriptions for Veterans with low back pain (LBP) persist despite the availability of PT, a lower medical risk treatment option. Patterns of treatment and subsequent healthcare utilization for Veterans with LBP are unknown. The purpose of this study was to evaluate the association of physical therapy (PT) and opioids and outcomes of spinal surgery and chronic opioid use for Veterans with incident LBP.

**Methods:**

We conducted a retrospective cohort study identifying Veterans with a new diagnosis of LBP using ICD codes from the Veterans Administration national database from 2012 to 2017. Veterans were classified into three treatment groups based on the first treatment received within 30 days of incident LBP: receipt of PT, opioids, or neither PT nor opioids. Outcomes, events of spinal surgery and chronic opioid use, were identified beginning on day 31 up to one year following initial treatment. We used propensity score matching to account for the potential selection bias in evaluating the associations between initial treatment and outcomes.

**Results:**

There were 373,717 incident cases of LBP between 2012 and 2017. Of those 28,850 (7.7%) received PT, 48,978 (13.1%) received opioids, and 295,889 (79.2%) received neither PT or opioids. Pain, marital status and the presence of cardiovascular, pulmonary, or metabolic chronic conditions had the strongest statistically significant differences between treatment groups. Veterans receiving opioids compared to no treatment had higher odds of having a spinal surgery (2.04, 99% CI: 1.67, 2.49) and progressing to chronic opioid use (11.8, 99% CI: 11.3, 12.3). Compared to Veterans receiving PT those receiving opioids had higher odds (1.69, 99% CI: 1.21, 2.37) of having spinal surgery and progressing to chronic opioid use (17.8, 99% CI: 16.0, 19.9).

**Conclusion:**

Initiating treatment with opioids compared to PT was associated with higher odds of spinal surgery and chronic opioid use for Veterans with incident LBP. More Veterans received opioids compared to PT as an initial treatment for incident LBP. Our findings can inform rehabilitation care practices for Veterans with incident LBP.

**Supplementary Information:**

The online version contains supplementary material available at 10.1186/s12913-023-09207-y.

## Background

Veterans with low back pain (LBP) returning from recent wars are 3 times more likely to develop chronic opioid use when compared to Veterans returning from recent wars without LBP. [1] Despite evidence indicating that opioids do not improve pain for patients with LBP, opioid prescriptions for LBP persist and subsequent substance abuse disorders and overdoses are increasing among Veterans and non-Veterans. [2–5] In 2017 the Department of Veterans Affairs (VA) and the Department of Defense issued practice guidelines for the medical management of LBP, consistent with non-VA LBP practice guidelines. [6–8] Among the recommended treatments for Veterans with LBP was the use of non-opioid medications such as nonsteroidal anti-inflammatory drugs (NSAIDs), as well as exercise and manual therapy, interventions performed by physical therapists. [6] Physical therapy (PT), a low medical risk intervention, has been shown to improve short term pain, function and disability for non-Veteran patients with LBP. [9, 10]

Common interventions used in the management of LBP include prescription opioids, spinal surgery, spinal injections and PT. [2, 6, 7, 9, 11, 12] PT is cost-effective and in non-VA users with LBP is associated with less subsequent healthcare utilization such as opioid use and spinal surgery. [11, 13−16] However, little is known about factors associated with treatment selection or the relationship between initial treatment selection and subsequent high risk healthcare utilization for Veterans with LBP. What is known is that a high percentage of Veterans experience LBP and a substantial proportion (23%) of Veterans returning from wars receive an opioid prescription. [1, 17] Overuse of opioids is of great concern considering the potential, fatal outcomes of long-term opioid use particularly among Veterans with concurrent mental health conditions. [18] Therefore, it is critical to identify interventions with lower medical risk for Veterans with LBP to maximize health and minimize unfavorable health consequences such as opioid misuse and more costly healthcare utilization.

The overall purpose of this research was to understand the association between initial treatment provided within the first 30 days after incident diagnosis of LBP and subsequent outcomes. We aimed to: (1) compare patterns of initial treatment (PT only, opioids only, and neither PT nor opioids); and (2) evaluate the association between initial treatment and receipt of spinal surgery and progression to chronic opioid use. The main hypothesis for this research is that Veterans receiving opioids as an initial intervention have higher odds of receiving spinal surgery and progressing to chronic opioid use compared to those receiving PT.

## Methods

This retrospective cohort study evaluated Veterans with an incident diagnosis of LBP in an outpatient setting between January 1, 2012 and December 31, 2017. This study protocol was conducted in accordance with the Declaration of Helsinki and ethical approval was obtained from our institution’s institutional review board (IRB). A waiver for receipt of informed consent was approved for this study.

### Data source

Data from the Department of Veterans Affairs Corporate Data Warehouse (CDW) within the (VA) Informatics and Computing Infrastructure (VINCI) was accessed. The CDW is a national database containing medical encounter data for health services rendered by the VA for Veterans of the United States Armed Forces. Data was derived from the outpatient, inpatient, procedure, pharmacy, and vital signs files. The files contain International Classification of Diseases (ICD) codes, Current Procedural Terminology (CPT) codes, drug names, pain intensity ratings and encounter dates. An encounter date identifies the receipt of a health service, for example, a physician visit or procedure.

### Cohort selection

#### Inclusion criteria

Veterans between the ages of 18-110 years with a new diagnosis of LBP occurring in the outpatient setting were identified using International Classification of Diseases, Ninth Revision, Clinical Modification (ICD-9-CM) and International Classification of Diseases, 10th Revision, (ICD10) codes indicating LBP. An additional file lists ICD diagnostic codes used to identify LBP [see Additional file 1]. LBP was defined by several musculoskeletal conditions used in previous LBP literature that represent thoraco-lumbar, lumbar, or lumbo-sacral disorders. [9] The incident date indicates the first physician encounter when a patient received a diagnosis of LBP. To validate the presence of LBP, a second ICD code representing LBP was identified within one year after the initial diagnosis and used as a confirmatory diagnosis code indicating that LBP was part of the ongoing medical history. Veterans were included in the analytic sample when evidence of a pain score from the vital signs file was available within 3 days of the incident diagnosis date.

#### Exclusion criteria

Veterans were excluded when CPT codes, ICD-9-CM codes, or ICD10 codes representing LBP, spinal surgery, or spinal injections were present one year prior to the incident date. This method was used to maximize the likelihood of Veterans having incident LBP. Veterans with evidence of opioid use one year prior to the incident date were also excluded. Veterans with evidence of a spinal trauma, pregnancy, or other medical condition where LBP symptoms may originate from a non-musculoskeletal condition one year prior to the incident date were excluded. Additional files list ICD diagnostic codes and CPT procedure codes, respectively, identifying exclusion criteria [see Additional files 2 and 3]. Veterans were excluded when it was not evident that the patient received VA medical care during the 6 months prior to the incident date.

#### Independent variables

**Fig. 1:**
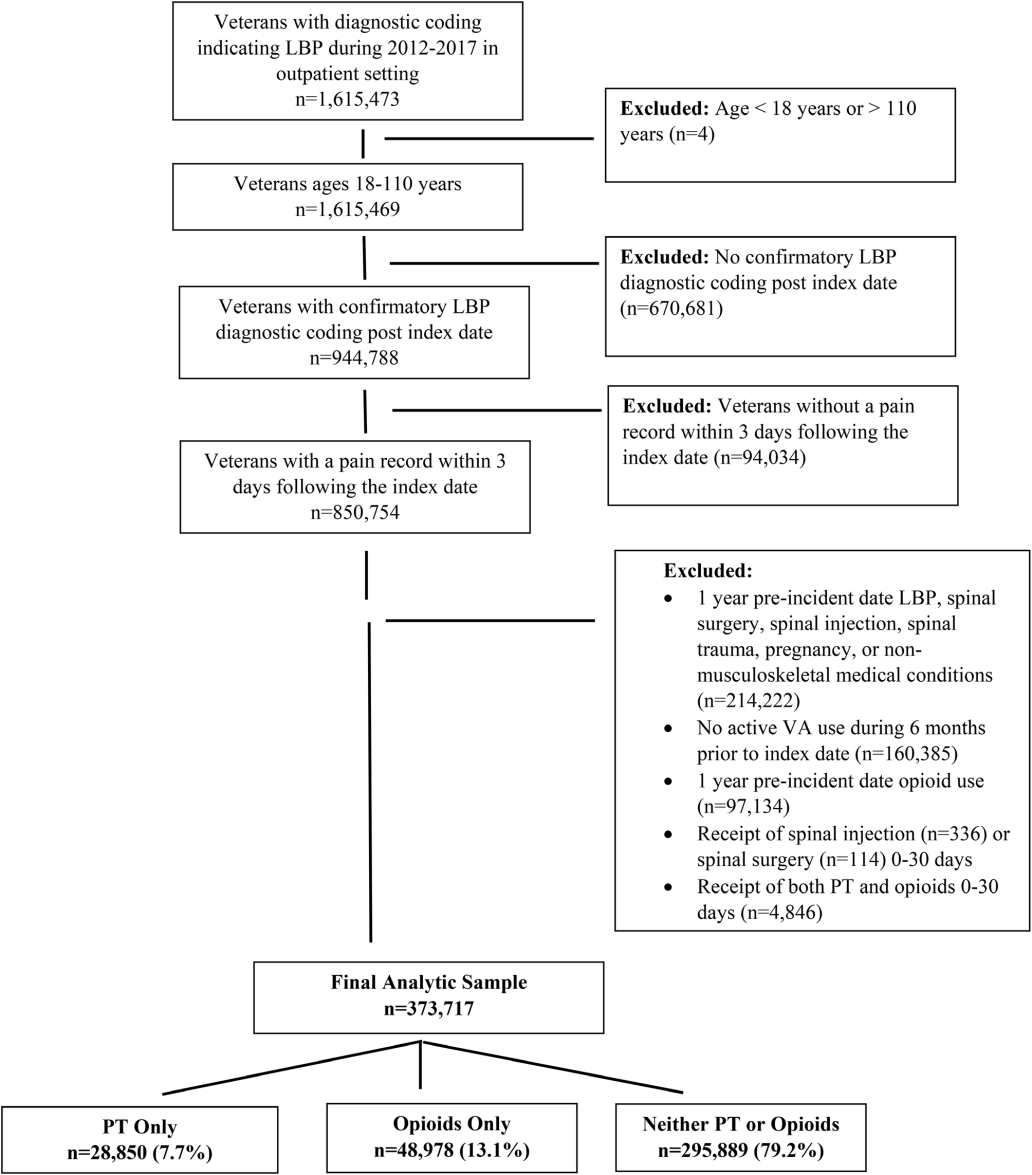
Flowchart for selection of study participants

The primary independent variable in this study was the first intervention received beginning on the incident date up to 30 days following the incident date. [9, 11, 16] We originally elected to evaluate the most common initial interventions received by patients with spinal conditions including: PT, opioids, spinal injection, spinal surgery and no treatment. [11, 12] However, because of the low prevalence of spinal injections and spinal surgery provided within the VA, Veterans receiving these interventions were excluded from the analytic sample (Fig. 1). Three mutually exclusive treatment groups were identified: PT, opioids, and no PT or opioids. Receipt of PT was identified from the procedures file using CPT codes. An Additional file lists CPT codes included in this study [see Additional file 3]. Opioids were identified from the pharmacy file using standardized VA classification codes. Opioids included: narcotic analgesics and narcotic analgesic combinations defined as any codeine, fentanyl, hydrocodone, hydromorphone, morphine, tramadol, oxycodone, acetaminophen with hydrocodone, acetaminophen with oxycodone, acetaminophen with codeine, or tramadol with acetaminophen. The remaining Veterans were classified into the no PT or opioids group.

#### Baseline characteristics

Baseline characteristics including age (years), sex (male, female), marital status (married, divorced/separated, single, widowed or unknown), race (white, black, other, unknown) and ethnicity (Hispanic, non-Hispanic, unknown) were identified from the patient demographic files at the incident date. The 18 chronic comorbidities included in the Functional Comorbidity Index (FCI) were selected from the outpatient and inpatient files using ICD-9-CM and ICD10 codes up to one year prior to the incident date. The comorbidities represent conditions most likely to impact functional mobility, a primary activity limitation for patients with LBP and included the following: osteoarthritis and/or rheumatoid arthritis, angina and/or ischemic heart disease, anxiety, asthma, congestive heart failure (CHF), chronic obstructive pulmonary disease (COPD), cerebral vascular accident (CVA) or transient ischemic attack, depression, diabetes, hearing impairment, acute myocardial infarction, obesity (body mass index greater than 30), osteoporosis, peripheral vascular disease (PVD), and visual impairment. [19] Additionally, we identified diagnoses of post-traumatic stress disorder (PTSD), psychosis, substance abuse disorders and alcohol use given the prevalence of these conditions for Veterans and the known impact on healthcare utilization outcomes for patients with LBP. [20, 21] An additional file lists mental health and behavioral health ICD codes [see Additional file 4]. Pain intensity was measured at the time of a clinical encounter as a subjective report of global pain using the Numeric Rating Scale (NRS).

#### Healthcare utilization outcome variables

Receipt of spinal surgery and progression to chronic opioid use, important outcomes for patients with spinal conditions, were identified beginning on day 31 following the initial LBP diagnosis up to one year. [22] Lumbar spinal surgery was identified using procedure and/or CPT codes from the procedures file. Chronic opioid use was defined as evidence of 10 or more opioid prescriptions filled within a one-year period or more than 120 days’ supply within a one-year period.

#### Statistical analyses

Baseline characteristics for Veterans across the treatment groups were compared using two methods. Age and baseline pain were compared using analysis of variance (ANOVA) with post hoc Tukey’s adjustment for multiple comparisons. The remaining baseline characteristics were compared using chi-square tests.

Before comparing the treatment groups, we accounted for selection bias manifested in significant differences in baseline characteristics between groups. We used propensity score weights, which we estimated using multinomial propensity scores (MNPS), a method designed for analyses with 3 or more treatment groups. [23] MNPS is an implementation of the toolkit for weighting and analysis of nonequivalent groups (twang package) for cases with more than two treatment conditions. [24] This approach is based on estimation of causal effect using inverse probability of treatment weights, estimated via generalized boosted models. The MNPS function estimates weights by repeated use of the “ps” function and comparing each treatment to the pooled sample of other treatments. [25] The main estimate is the average treatment effect (ATE), which represents the difference if all participants in the population of interest had been assigned to a particular treatment group compared to assignment to another single treatment group. The MNPS method establishes a balance on all baseline variables with significant differences by treatment group, and generates propensity weights for each observation using generalized boosted models. This process results in weighted baseline variables with similar distributions across groups. We included the following baseline variables: age, baseline pain, sex, marital status, race, ethnicity, comorbid conditions from the FCI, post-traumatic stress disorder, psychosis, substance abuse disorders and alcohol use. We evaluated the validity of the propensity models using graphical assessment of balance by plotting the absolute standardized mean differences in covariates between treatment groups. The demographic characteristics of propensity weighted groups were compared using t-tests and chi-squared tests.

We used quasi-binomial generalized linear model with propensity score matching to compare the treatment effect for two separate outcomes: receipt of spinal surgery and progression to chronic opioid use. Odds ratio estimates were obtained for each treatment group pair. To adjust for multiple comparisons, we used Tukey’s method (implemented with the glht function in R multcomp package). The model compares Veterans in the opioid group to the no PT or opioid group, the PT group to the no PT or opioid group, and the PT group compared to the opioid group.

Analyses were performed using SAS Enterprise-G 7.15 and R 4.0.4.

## Results

Among the 1,615,473 Veterans with a documented LBP diagnosis between 2012 and 2017, there were 373,717 who met the study’s inclusion criteria (Fig. 1). During the first 30 days following incident diagnosis of LBP, 28,850 (7.7%) received PT, 48,978 (13.1%) received opioids, and 295,889 (79.2%) did not receive a study treatment (Table 1). Veterans receiving PT as an initial treatment were younger (52.8, SD 17.9) compared to those receiving opioids (54.2, SD 16.6) and those not receiving PT or opioids (53.2, SD 17.4). Veterans receiving opioids as an initial treatment experienced greater pain (5.6, SD 3.0) compared to those receiving PT (4.2, SD 3.0) and those not receiving PT or opioids (3.8, SD 3.1) (Table 1). In the overall sample most were white and married, non-Hispanic males.

**Table 1:** Sample Demographics and Characteristics by Group (n = 373,717)

	PT Only N = 28,850	Opioids Only N = 48,978	No PT or Opioids N = 295,889	
	Mn (SD)	Mn (SD)	Mn (SD)	ANOVA p
Age	52.8 (17.9)	54.2 (16.6)	53.2 (17.4)	< 0.0001*
Baseline Pain	4.2 (3.0)	5.6 (3.0)	3.8 (3.1)	< 0.0001*
	**N (%)**	**N (%)**	**N (%)**	**Chi-square p**
**Sex**				**< 0.0001**
Male	25,505 (88.4)	44,528 (90.9)	265,096 (89.6)	
**Marital Status**				**< 0.0001**
Divorced/Separated	7018 (24.3)	14,580 (29.8)	73,550 (24.9)	
Married	15,526 (53.8)	25,619 (52.2)	164,179 (55.5)	
Single	4896 (16.9)	6295 (12.9)	44,073 (14.9)	
Widowed	1168 (4.1)	2165 (4.4)	11,188 (3.8)	
Other (Unknown)	242 (0.8)	319 (0.7)	2899 (1.0)	
**Race**				**< 0.0001**
White	20,033 (69.4)	35,265 (72.0)	204,675 (69.2)	
Black	5838 (20.2)	9120 (18.6)	58,739 (19.9)	
Other**	1360 (4.7)	1752 (3.6)	13,240 (4.5)	
Unknown	1619 (5.6)	2841 (5.8)	19,235 (6.5)	
**Ethnicity**				**< 0.0001**
Hispanic	2225 (7.7)	3577 (7.3)	26,251 (8.9)	
Non-Hispanic	25,623 (88.8)	43,712 (89.3)	258,668 (87.4)	
Unknown	1002 (3.5)	1689 (3.5)	10,970 (3.7)	
**Comorbid Conditions** **Musculoskeletal**				
Arthritis (Rheumatoid/Osteoarthritis)	2864 (10.0)	5006 (10.2)	25,868 (8.7)	**< 0.0001**
Osteoporosis	144 (0.5)	258 (0.5)	1259 (0.4)	**0.0027**
**Cardiovascular**				
Angina/Ischemic Heart Disease	2159 (7.5)	4144 (8.5)	19,815 (6.7)	**< 0.0001**
Cerebral Vascular Accident/Transient Ischemic Attack	741 (2.6)	1001 (2.0)	5474 (1.9)	**< 0.0001**
Congestive Heart Failure	446 (1.6)	1093 (2.2)	3693 (1.3)	**< 0.0001**
Myocardial Infarction (acute)	50 (0.2)	107 (0.2)	381 (0.1)	**< 0.0001**
Peripheral Vascular Disease	466 (1.6)	927 (1.9)	3983 (1.4)	**< 0.0001**
**Pulmonary**				
Asthma	882 (3.1)	1404 (2.9)	7512 (2.5)	**< 0.0001**
Chronic Obstructive Pulmonary Disease (COPD)	487 (1.7)	963 (2.0)	3662 (1.2)	**< 0.0001**
**Metabolic**				
Diabetes (Types I and II)	3958 (13.7)	7239 (14.8)	36,238 (12.3)	**< 0.0001**
Gastrointestinal (upper-ulcer, hernia, reflux)	745 (2.6)	868 (1.8)	4783 (1.6)	**< 0.0001**
Obesity (BMI > 30)	3408 (11.8)	5274 (10.8)	29,028 (9.8)	**< 0.0001**
**Mental Health**				
Anxiety	6066 (21.0)	9103 (18.6)	54,253 (18.3)	**< 0.0001**
Depression	2997 (10.4)	3867 (7.9)	23,097 (7.8)	**< 0.0001**
Post-traumatic Stress Disorder	4020 (13.9)	5849 (11.9)	35,621 (12.0)	**< 0.0001**
Psychosis	468 (1.6)	709 (1.5)	4131 (1.4)	**0.0071**
**Other**				
Hearing Impairment	3419 (11.9)	4478 (9.1)	29,499 (10.0)	**< 0.0001**
Visual Impairment	240 (0.8)	377 (0.8)	2321 (0.8)	0.6205
Neurological Disease (Multiple Sclerosis/Parkinson’s)	157 (0.5)	171 (0.4)	1148 (0.4)	**< 0.0001**
Substance Use Disorder **(excluding opioid use in previous year)	1410 (4.9)	1779 (3.6)	10,439 (3.5)	**< 0.0001**
Alcohol Use	2362 (8.2)	3211 (6.6)	18,599 (6.3)	**< 0.0001**

*comparisons between all groups for age and pain intensity were statistically significantly different using the post hoc Tukey’s adjustment. **Other race category includes American Indian, Islander, and Asian

The prevalence of comorbid conditions for Veterans receiving PT, opioids, and no PT or opioids are listed within Table 1. There were statistically significant differences between groups in the prevalence of all comorbidities except visual impairment. In the overall sample, mental health and metabolic disorders were the most prevalent comorbid conditions.

**Fig. 2:**
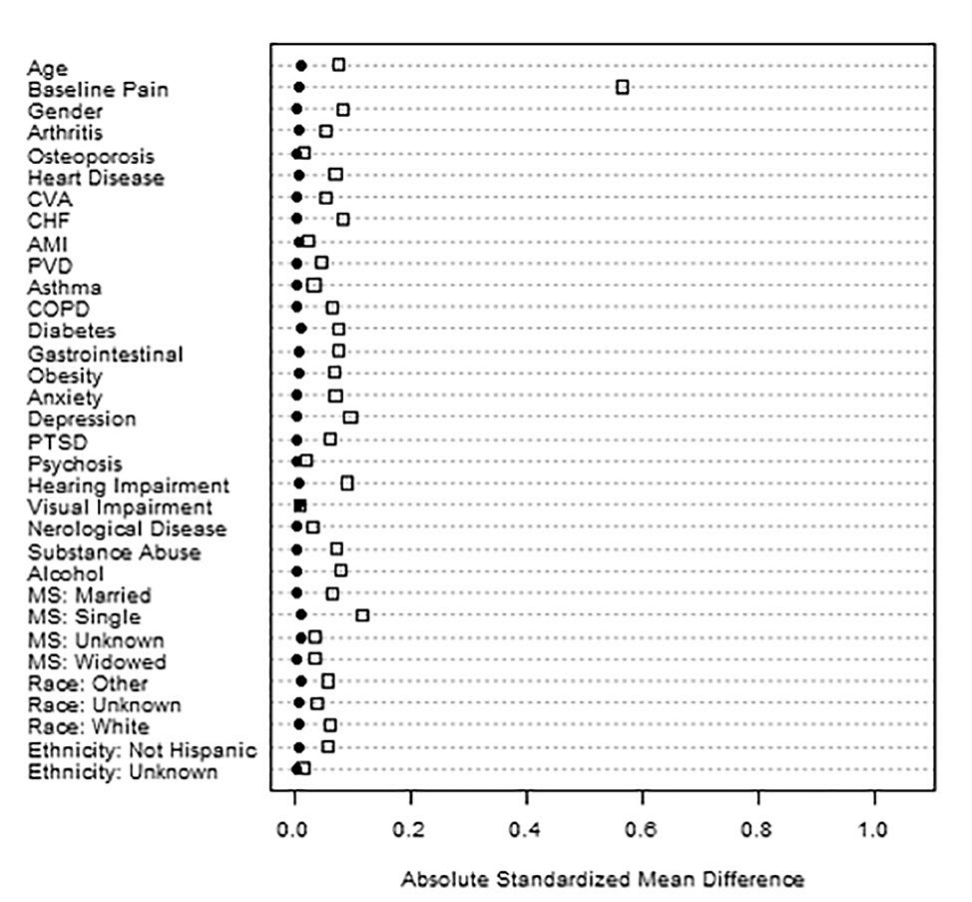
Maximum Absolute Standardized Mean Differences Before and After Propensity Matching Footnotes: Maximum of paired standardized effect sizes between treatment groups; Squares indicate before propensity matching; Circles indicate after propensity matching

After propensity score matching all baseline differences between treatment groups were balanced. Visual assessment of propensity matching indicated a good balance between groups (Fig. 2). Figure 2 shows the maximum value of absolute standardized mean differences for covariates between the treatment groups. All differences were less than 0.02 after propensity matching, indicating a good balance between treatment groups.

The unadjusted and adjusted prevalence for spinal surgery and chronic opioid use by treatment group are listed in Table 2. There was a greater prevalence of having spinal surgery and progressing to chronic opioid use for Veterans receiving opioids as an initial intervention compared to Veterans receiving PT or neither PT nor opioids.

**Table 2:** Unadjusted and Adjusted Prevalence of Outcomes by Treatment Group

Unadjusted*	**PT Only** **N = 28,850**	**Opioids Only** **N = 48,978**	**No PT or Opioids** **N = 295,889**
	**N (%)**	**N (%)**	**N (%)**
Spinal Surgery	103 (0.4)	374 (0.8)	873 (0.3)
Chronic Opioid Use	801 (2.8)	16,683 (34.1)	11,987 (4.1)
Adjusted**	**PT Only** **N = 27,502.81**	**Opioids Only** **N = 37,589.85**	**No PT or Opioids** **N = 293,135.85**
	%	%	%
Spinal Surgery	0.4%	0.6%	0.3%
Chronic Opioid Use	2.8%	34.2%	4.2%

*Unadjusted indicates before propensity score matching; **Adjusted indicates after propensity score matching; effective sample sizes are reported for groups after propensity score matching

Results of quasi-binomial regressions of having a spinal surgery and progressing to chronic opioid use by treatment group are provided in Table 3. After propensity score matching, Veterans receiving opioids as an initial intervention (compared to no opioids or PT) had 2.04 (99%CI: 1.67, 2.49) times higher odds of progressing to spinal surgery, and 11.8 (11.3, 12.3) times higher odds of progressing to chronic opioid use. While the treatment group of Veterans receiving PT (compared to no opioids or PT) was not associated with spinal surgery, the treatment group had 0.66 (0.59, 0.74) times lower odds of progressing to chronic opioid use. Additionally, Veterans receiving opioids (compared to PT) had 1.69 (1.21, 2.37) times higher odds of progressing to spinal surgery and 17.8 (16.0, 19.9) times higher odds of progressing to chronic opioid use.

**Table 3:** Association Between Initial Treatment and Spinal Surgery or Chronic Opioid Use*

	Spinal Surgery		Chronic Opioid Use	
	AOR* (99%CI)	Tukey adjusted p	AOR (99%CI)	Tukey adjusted p
Treatment effects				
Opioid only vs. none	2.04 (1.67, 2.49)	< 0.0001	11.8 (11.3, 12.3)	< 0.0001
PT only vs. none	1.20 (0.89, 1.64)	0.1790	0.66 (0.59, 0.74)	< 0.0001
Opioid only vs. PT only	1.69 (1.21, 2.37)	< 0.0001	17.8 (16.0, 19.9)	< 0.0001
Cox-Snell R^2^	0.000		0.156	

*Chronic Opioid Use: 10 or more opioid prescriptions filled within a 1-year period or more than 120 days’ supply within a 1-year period beginning on day 31 following incident diagnosis of LBP. Quasi-binomial generalized linear models were adjusted for propensity scoring by age, baseline pain, sex, marital status, race, ethnicity and comorbidities

## Discussion

Veterans receiving opioids as an initial treatment for LBP had 17.8 higher odds of progressing to chronic opioid use within a year compared to those receiving PT in the propensity matched models. These findings are consistent with our hypothesis as well as a previous study that identified the association between early opioid prescribing and subsequent long-term opioid use for patients with noncancer pain. [26] Additionally, the results support LBP guideline recommendations against early opioid prescription as an initial treatment for LBP as it may lead to chronic opioid use and fatal consequences. [6–8].

Our study also found that Veterans receiving PT as an initial treatment compared to Veterans not receiving PT or opioids had 0.66 lower odds of progressing to chronic opioid use. This finding is consistent with a study from the Military Health System that reported receipt of early PT reduced the likelihood of opioids as well as surgery and other costly health services. [27] Interestingly, our study did not find a statistically significant relationship between initial PT intervention and spinal surgery. We found that spinal surgery was seldom utilized by Veterans within the VA, with fewer than 1% of Veterans across treatment groups receiving spinal surgery. Other population-based studies identified slightly higher proportions (1.3%, 3.1%) of patients with LBP receiving spinal surgery after PT intervention. [11, 27] The low prevalence of spinal surgery may have contributed to the lack of association between PT and spinal surgery. Additionally, while our study focused on health services delivered through the VA, we acknowledge that Veterans may have received care, including spinal surgery, outside of the VA.

This research is one of the first to describe initial treatment selections and to evaluate the associations between receiving early PT or opioids and healthcare outcomes among United States Veterans with incident LBP. One of the most striking findings from our study was that most (79.2%) Veterans with incident LBP did not receive PT or opioids. Further, PT utilization was far less frequent than opioid utilization (7.7% vs. 13.1%). The proportion of Veterans who received PT in our study was lower than reported in other studies (12–20%) after a new onset of a spinal condition. [11, 22, 28] A possible explanation for the reduced rate of PT utilization in our study was that Veterans may have selected other interventions for LBP such as acupuncture, yoga, or chiropractic care, or received care outside of the VA. [29] We were not able to evaluate receipt of alternate interventions and services rendered outside of the VA. Future studies evaluating both VA and non-VA health services may provide a more comprehensive description of healthcare utilization among Veterans with incident LBP.

### Limitations

Our findings are limited to United States Veterans receiving care through the VA. Like any observational study where interventions are not randomly assigned there are risks of selection bias and potential confounding. We acknowledge that referral to and utilization of interventions may be influenced by the intensity of LBP. We implemented propensity matching analysis to mitigate potential selection bias and confounding based on the differences identified between baseline variables, in particular baseline pain. We recognize that propensity score matching can be limited due to the assumption that this method matches on observable measures. While we controlled for many variables that could potentially confound the relationship between treatment groups and the study outcomes, there is the possibility that other confounding factors were not measured or available within the data and potentially contributed to the large effect of chronic opioid use. One example is psychosocial behaviors such as, pain catastrophizing and social isolation which have been linked to higher pain and functional limitation for patients with LBP and can influence patients to seek pain management with opioids. [30].

We recognize that the data used for this analysis is dated and that it would be beneficial to repeat analyses with current data before suggesting major practice plan or policy changes.

Our sample included opioid naïve patients. Veterans were excluded if there was evidence within the data of an opioid prescription one year prior to the LBP incident date. Our study identified large effect sizes for Veterans who progressed to chronic opioid use. One explanation is that Veterans may have been using opioids obtained outside of the VA or illicitly during the initial treatment period. While the LBP guidelines do not support the use of opioids for a new LBP episode Veterans may have sought pain management elsewhere. We did not have access to opioid utilization outside the VA or to illicit use of opioids by Veterans.

More Veterans did not receive either PT or opioids during the first 30 days following incident diagnosis. While our initial treatment time frame reflects current practice patterns we recognize that Veterans may have received either PT or opioids at a later time point, after 30 days up to one year. We did not track Veterans receiving treatment between 31 days up to one year after the incident date, but recognize that unobserved treatments received during this time may have influenced the large effect size for Veterans progressing to chronic opioid use.

Finally, to minimize the potential for misclassifying a Veteran with LBP when they did not have LBP, we included only those Veterans with a second LBP diagnosis code over a one-year period. The presence of an additional diagnosis code representing LBP increases the likelihood that the Veteran had ongoing care for LBP at a subsequent medical encounter within the VA.

## Conclusion

Our study found that initiating treatment with opioids during the first 30 days was associated with higher odds of having a spinal surgery and progressing to chronic opioid use. We found that a higher percentage of Veterans received opioids during the first 30 days as compared to PT, a non-invasive and effective treatment at decreasing symptoms and improving function. However, most Veterans with incident LBP received neither PT or opioids within the first 30 days after diagnosis. Findings can inform rehabilitative care prescription for Veterans with incident LBP.

## Electronic supplementary material

Below is the link to the electronic supplementary material.

Supplementary Material 1

Supplementary Material 2

Supplementary Material 3

Supplementary Material 4

Supplementary Material 5

## Data Availability

The data that support the findings of this study are available from the Department of Veterans Affairs but restrictions apply to the availability of these data, which were used under license for the current study, and so are not publicly available. Data may be available from the authors upon reasonable request and with permission of the Department of Veterans Affairs.

## Declarations

### Authors’ information

CS is an Assistant Professor and health services researcher in the Department of Physical Therapy at MGH Institute of Health Professions. CS has over 15 years of clinical experience providing physical therapy care to patients with musculoskeletal conditions including 9 years of managerial experience for a private outpatient practice. CS has focused her research on the utilization of physical therapy and the association of this lower medical risk intervention on healthcare outcomes for patients with musculoskeletal conditions including spinal conditions such as low back pain.

## List of abbreviations

ANOVA: analysis of variance
ATE: average treatment effect
CDW: Corporate Data Warehouse
CHF: congestive heart failure
COPD: chronic obstructive pulmonary disease
CPT: Current Procedural Terminology
CVA: cerebral vascular accident
FCI: Functional Comorbidity Index
ICD: International Classification of Diseases
ICD-9-CM: International Classification of Diseases, Ninth Revision, Clinical Modification
ICD10: International Classification of Diseases, 10th Revision
LBP: low back pain
MNPS: multinomial propensity scores
NRS: numeric pain rating scale
NSAIDs: non-steroidal anti-inflammatory drugs
PT: physical therapy
PTSD: post-traumatic stress disorder
PVD: peripheral vascular disease
VA: Department of Veterans Affairs
VINCI: Department of Veterans Affairs Informatics and Computing Infrastructure
