# Supplementary material for: Initial treatment approaches and healthcare utilization among veterans with low back pain: a propensity score analysis
Source: BMC Health Serv Res. 2023 Mar 21;23:275. doi: 10.1186/s12913-023-09207-y (PMC10029316; doi:10.1186/s12913-023-09207-y)
Supplement: Supplementary file 1 — Supplementary Material 1 [file 12913_2023_9207_MOESM1_ESM.docx]

**Additional file 1**

**Table A1: ICD Codes Used to Identify Low Back Pain**

| **ICD-9-CM Code** | **Description** | **ICD-9-CM Code** | **Description** |
| --- | --- | --- | --- |
| 720.0 | Sacroilitis | 724.3 | Sciatica |
| 721.3 | Lumbosacral spondylosis w/o myelopathy | 724.4 | Thoracic or lumbosacral neuritis or radiculitis, unspecified |
| 721.4 | Thoracic or lumbar spondylosis with myelopathy | 724.5 | Backache, unspecified |
| 721.42 | Lumbar spondylosis without myelopathy | 724.6 | Disorders of sacrum |
| 722.1 | Displacement of thoracic or lumbar intervertebral disc w/o myelopathy | 737.2 | Lordosis (acquired), (postural) |
| 722.10 | Displacement of lumbar inter-vertebral disc w/o myelopathy | 737.29 | Lordosis acquired other |
| 722.32 | Schmorl's nodes lumbar region | 737.42 | Curvature of the spine associated with other conditions, lordosis |
| 722.5 | Degeneration of thoracic or lumbar intervertebral disc | 739.3 | Nonallopathic lesions, lumbar region |
| 722.51 | Degeneration of thoracic or thoracolumbar intervertebral disc | 739.4 | Nonallopathic lesions, sacral region |
| 722.52 | Degeneration of lumbar or lumbosacral intervertebral disc | 756.11 | Spondylosis, lumbosacral region |
| 722.73 | Intervertebral disc disorder w/ myelopathy, lumbar region | 846 | Sprains and strains of sacroiliac region |
| 722.93 | Other and unspecified disc disorder, lumbar region | 846.0 | Sprains and strains-lumbosacral (joint) (ligament) |
| 724.01 | Spinal stenosis-thoracolumbar | 846.1 | Sprains and strains-sacroiliac ligament |
| 724.02 | Spinal stenosis, other than cervical-lumbar region | 846.2 | Sprains and strains-sacrospinus (ligament) |
| 724.03 | Spinal stenosis with neurogenic claudication-lumbar region | 846.3 | Sprains and strains-sacrotuberous (ligament) |
| 724.2 | Lumbago, low back pain, low back syndrome | 846.8, 846.9 | Sprains and strains-other specified and unspecified sites of sacroiliac region |
| **ICD 10 Code** | **Description** | **ICD 10 Code** | **Description** |
| M40.05 | Postural kyphosis, thoracolumbar region | M48.062 | Spinal stenosis, lumbar region, with neurogenic claudication |
| M40.15 | Other secondary kyphosis, thoracolumbar region | M48.07 | Spinal stenosis, lumbosacral region |
| M40.205 | Other and unspecified kyphosis, thoracolumbar region | M48.15 | Ankylosing hyperostosis, thoracolumbar region |
| M40.295 | Other kyphosis, thoracolumbar region | M48.16 | Ankylosing hyperostosis, lumbar region |
| M40.35 | Flatback syndrome, thoracolumbar region | M48.17 | Ankylosing hyperostosis, lumbosacral region |
| M40.36 | Flatback syndrome, lumbar region | M48.25 | Kissing spine, thoracolumbar spine |
| M40.37 | Flatback syndrome, lumbosacral region | M48.26 | Kissing spine, lumbar spine |
| M40.45 | Postural lordosis, thoracolumbar region | M48.27 | Kissing spine, lumbosacral spine |
| M40.46 | Postural lordosis, lumbar region | M48.8X5 | Other specified spondylopathies, thoracolumbar region |
| M40.47 | Postural lordosis, lumbosacral region | M48.8X6 | Other specified spondylopathies lumbar region |
| M40.55 | Lordosis, unspecified, thoracolumbar region | M48.8X7 | Other specified spondylopathies, lumbosacral region |
| M40.56 | Lordosis, unspecified, lumbar region | M49.85 | Spondylopathy in diseases classified elsewhere, thoracolumbar region |
| M40.57 | Lordosis, unspecified, lumbosacral region | M49.86 | Spondylopathy in diseases classified elsewhere, lumbar region |
| M41.25 | Other idiopathic scoliosis, thoracolumbar region | M49.87 | Spondylopathy in diseases classified elsewhere, lumbosacral region |
| M41.26 | Other idiopathic scoliosis, lumbar region | M51.05 | Intervertebral disc disorders w/ myelopathy, thoracolumbar region |
| M41.27 | Other idiopathic scoliosis, lumbosacral region | M51.06 | Intervertebral disc disorders w/ myelopathy, lumbar region |
| M41.45 | Neuromuscular scoliosis, thoracolumbar region | M51.15 | intervertebral disc disorders w/ radiculopathy, thoracolumbar region |
| M41.46 | Neuromuscular scoliosis, lumbar region | M51.16 | intervertebral disc disorders w/ radiculopathy, lumbar region |
| M41.47 | Neuromuscular scoliosis, lumbosacral region | M51.17 | intervertebral disc disorders w/ radiculopathy, lumbosacral region |
| M41.55 | Other secondary scoliosis, thoracolumbar region | M51.25 | Other intervertebral disc displacement, thoracolumbar region |
| M41.56 | Other secondary scoliosis, lumbar region | M51.26 | Other intervertebral disc displacement, lumbar region |
| M41.57 | Other secondary scoliosis, lumbosacral region | M51.27 | Other intervertebral disc displacement, lumbosacral region |
| M41.85 | Other forms of scoliosis, thoracolumbar region | M51.35 | Other intervertebral disc degeneration, thoracolumbar region |
| M41.86 | Other forms of scoliosis, lumbar region | M51.36 | Other intervertebral disc degeneration, lumbar region |
| M41.87 | Other forms of scoliosis, lumbosacral region | M51.37 | Other intervertebral disc degeneration, lumbosacral region |
| M42.15 | Spinal osteochondrosis, Adult, thoracolumbar region | M51.45 | Schmorl’s nodes thoracolumbar region |
| M42.16 | Spinal osteochondrosis, Adult, lumbar region | M51.46 | Schmorl’s nodes lumbar region |
| M42.17 | Spinal osteochondrosis, Adult, lumbosacral region | M51.47 | Schmorl’s nodes lumbosacral region |
| M43.05 | Spondylosis, thoracolumbar region | M51.85 | Other intervertebral disc disorders, thoracolumbar region |
| M43.06 | Spondylosis, lumbar region | M51.86 | Other intervertebral disc disorders, lumbar region |
| M43.07 | Spondylosis, lumbosacral region | M51.87 | Other intervertebral disc disorders, lumbosacral region |
| M46.05 | Spinal enthesopathy, thoracolumbar region | M53.2 | Instability, dislocation, lumbosacral/sacroiliac |
| M46.06 | Spinal enthesopathy, lumbar region | M53.2X5 | Spinal instability, thoracolumbar region |
| M46.07 | Spinal enthesopathy, lumbosacral region | M53.2X6 | Spinal instability, lumbar region |
| M46.1 | Sacroilitis, not elsewhere classifed | M53.2X7 | Spinal instability, lumbosacral region |
| M46.45 | Discitis,unspecified,thoracolumbar region | M53.85 | Other specified dorsopathies, thoracolumbar region |
| M46.46 | Discitis,unspecified, lumbar region | M53.86 | Other specified dorsopathies, lumbar region |
| M46.47 | Discitis,unspecified,lumbosacral region | M53.87 | Other specified dorsopathies, lumbosacral region |
| M46.85 | Other specified inflammatory spondylopathies, thoracolumbar region | M54.15 | Radiculopathy, thoracolumbar region |
| M46.86 | Other specified inflammatory spondylopathies, lumbar region | M54.16 | Radiculopathy, lumbar region |
| M46.87 | Other specified inflammatory spondylopathies, lumbosacral region | M54.17 | Radiculopathy, lumbosacral region |
| M46.95 | Unspecified inflammatory spondylopathy, thoracolumbar region | M54.18 | Radiculopathy, sacral and sacrococcygeal region |
| M46.96 | Unspecified inflammatory spondylopathy, lumbar region | M54.30 | Sciatica, unspecified site |
| M46.97 | Unspecified inflammatory spondylopathy, lumbosacral region | M54.31 | Sciatica, unspecified site, right side |
| M47.015 | Spondylosis, thoracolumbar region | M54.32 | Sciatica, unspecified site, left side |
| M47.016 | Spondylosis, lumbar region | M54.40 | Lumbago with sciatica, unspecified site |
| M47.15 | Spondylosis, thoracolumbar region | M54.41 | Lumbago with sciatica, unspecified site, right side |
| M47.16 | Spondylosis, lumbar region | M54.42 | Lumbago with sciatica, unspecified site, left side |
| M47.25 | Other spondylosis with radiculopathy, thoracolumbar region | M54.5 | Lumbago/low back pain |
| M47.26 | Other spondylosis with radiculopathy, lumbar region | M54.9 | Backache (postural); other dorsalgia; pain, spine, back |
| M47.27 | Other spondylosis with radiculopathy, lumbosacral region | S33.5XXA | Sprain of ligaments of lumbar spine, initial encounter |
| M47.815 | Other spondylosis w/o myelopathy or radiculopathy, thoracolumbar region | S33.6XXA | Sprain of sacroiliac joint, initial encounter |
| M47.816 | Other spondylosis w/o myelopathy or radiculopathy, lumbar region | S33.8XXA | Sprain of other parts of lumbar spine and pelvis, initial encounter |
| M47.817 | Other spondylosis w/o myelopathy or radiculopathy, lumbosacral region | S33.9XXA | Sprain of unspecified parts of lumbar spine and pelvis, initial encounter |
| M47.895 | Other spondylosis, thoracolumbar region | S34.21XA | Injury of nerve root of lumbar spine, initial encounter |
| M47.896 | Other spondylosis, lumbar region | S34.22XA | Injury of nerve root of sacral spine, initial encounter |
| M47.897 | Other spondylosis, lumbosacral region | S39.002A | Unspecified injury of muscle, fascia and tendon of lower back, initial encounter |
| M48.05 | Spinal stenosis, thoracolumbar | S39.012A | Strain of muscle, fascia and tendon of lower back, initial encounter |
| M48.06 | Spinal stenosis, lumbar region | S39.013A | Strain of muscle, fascia and tendon of pelvis, initial encounter |
| M48.061 | Spinal stenosis, lumbar region, w/o neurogenic claudication |  | |
