# Supplementary material for: Initial treatment approaches and healthcare utilization among veterans with low back pain: a propensity score analysis
Source: BMC Health Serv Res. 2023 Mar 21;23:275. doi: 10.1186/s12913-023-09207-y (PMC10029316; doi:10.1186/s12913-023-09207-y)
Supplement: Supplementary file 2 — Supplementary Material 2 [file 12913_2023_9207_MOESM2_ESM.docx]

**Additional file 2**

**Table A2: ICD Codes Identifying Conditions Used as Exclusion Criteria**

| **ICD-9-CM Code** | **Description** | **ICD-9-CM Code** | **Description** |
| --- | --- | --- | --- |
| 592.00-592.99 | Calculus of kidney | 805.x–809.x, 820.x–821.x, 733.13–733.15, or 733.96–733.98 | Fracture or stress fracture of the spine or pelvis |
| 574.x | gallbladder stones | 344.6x | Cauda equine syndrome |
| 599.0 | Urinary tract infection, site not specified | 730.xx | Osteomyelitis, periostitis, and other infections involving bone |
| V13.02 | Urinary (tract) infection | 731.3 | Major osseous deficit |
| 140.xx – 239.xx | Neoplasms | 342.00-342.02, 342.10-342.12, 342.80-342.82, 342.90-342.92 | Hemiplegia, hemiparesis |
| 344.1 | Paraplegia | 344.30-344.32, 344.40-344.42, 344.5 | Monoplegia |
| 344.00-344.04, -.09 | Quadraplegia | V46.3 | Wheelchair dependence |
| **ICD-10 Code** | **Description** | **ICD-10 Code** | **Description** |
| N20.0 | Calculus of kidney | S32.00xx | Fracture or stress fracture of the spine or pelvis |
| N20.1 | Calculus of ureter | G83.4 | Cauda equine syndrome |
| N20.2 | Calculus of kidney with calculus of ureter | M45.5-9 | Ankylosing spondylitis |
| N20.9 | Urinary calculus, unspecified | M46.05-08 | Other inflammatory spondylopathies |
| N21.0 | Calculus in bladder | M46.25-28 | Osteomyelitis of vertebra |
| N21.1 | Calculus in urethra | M46.35-38 | Infection of vertebra |
| N21.8 | Other lower urinary tract calculus | C41.2 | Malignant neoplasm of vertebral column |
| N21.9 | Calculus of lower urinary tract, unspecified | C41.4 | Malignant neoplasm of pelvic bones, sacrum and coccyx |
| N39.0 | Urinary tract infection, site not specified | M89.70 | Major osseous defect, unspecified site |
| K80.00 | Cholelithiasis, without obstruction | G81.00-G81.04, G81.10-G81.14, G81.90-G81.94 | Hemiplegia, hemiparesis |
| K80.01 | Cholelithiasis, with obstruction | G82.20-G82.22 | Paraplegia |
| K80.10-13, K80.18-19 | Cholelithiasis disorders | G82.50-G82.54 | Quadraplegia |
| Z99.3 | Wheelchair dependence |  | |
