# Supplementary material for: Initial treatment approaches and healthcare utilization among veterans with low back pain: a propensity score analysis
Source: BMC Health Serv Res. 2023 Mar 21;23:275. doi: 10.1186/s12913-023-09207-y (PMC10029316; doi:10.1186/s12913-023-09207-y)
Supplement: Supplementary file 3 — Supplementary Material 3 [file 12913_2023_9207_MOESM3_ESM.docx]

**Additional file 3**

| **Table A3: CPT-4 Codes Identifying Procedures** | | |
| --- | --- | --- |
| **Procedure** | **CPT-4 Code** | **Description** |
| Physical Therapy | 97161 | Physical therapy evaluation: low complexity, requiring these components: |
|  | 97162 | Physical therapy evaluation: moderate complexity, requiring these components: |
|  | 97163 | Physical therapy evaluation: high complexity, requiring these components: |
|  | 97164 | Re-evaluation of physical therapy established plan of care, requiring these components: |
|  |  | **Modalities** |
|  | G0281 | Electrical stimulation (unattended), to one or more areas, for chronic stage III and stage IV ulcers |
|  | G0282 | Electrical stimulation, (unattended), to one or more areas, for wound care other than described in G0281 |
|  | G0283 | Electrical stimulation (unattended), to one or more areas for indication(s) other than wound care, as part of a therapy plan of care |
|  | 90901 | Biofeedback training by any modality |
|  | 97010 | Hot/Cold Packs |
|  | 97012 | Application of a modality to one or more areas; traction, mechanical |
|  | 97014 | Electrical Stimulation (unattended) |
|  | 97016 | vasopneumatic devices |
|  | 97018 | paraffin bath |
|  | 97022 | whirlpool |
|  | 97024 | Diathermy (eg, microwave) |
|  | 97026 | Infrared |
|  | 97028 | Ultraviolet |
|  | 97032 | Application of a modality to one or more areas; electrical stimulation (manual), each 15 minutes |
|  | 97033 | iontophoresis, each 15 minutes |
|  | 97034 | contrast baths, each 15 minutes |
|  | 97035 | ultrasound, each 15 minutes |
|  | 97036 | Hubbard tank, each 15 minutes |
|  | 97039 | Unlisted modality (specify type and time if constant attendance) |
|  |  | **Therapeutic Procedures** |
|  | 97110 | Therapeutic procedure, one or more areas, each 15 minutes; therapeutic exercises to develop strength and endurance, range of motion and flexibility |
|  | 97112 | neuromuscular reeducation of movement, balance, coordination, kinesthetic sense, posture, and/or proprioception for sitting and/or standing activities |
|  | 97113 | aquatic therapy with therapeutic exercises |
|  | 97116 | gait training (includes stair climbing) |
|  | 97124 | massage, including effleurage, petrissage and/or tapotement (stroking, compression, percussion) |
|  | 97139 | Unlisted therapeutic procedure (specify) |
|  | 97140 | Manual therapy techniques (eg, mobilization/manipulation, manual lymphatic drainage, manual traction), one or more regions, each 15 minutes |
|  | 97150 | Group Therapy |
|  | 97530 | Therapeutic activities, direct (one-on-one) patient contact (use of dynamic activities to improve functional performance), each 15 minutes |
|  | 97533 | Sensory integrative techniques to enhance sensory processing and promote adaptive responses to environmental demands, direct (one-on-one) patient contact, each 15 minutes |
|  | 97535 | Self-care/home management training (eg, activities of daily living (ADL) and compensatory training, meal preparation, safety procedures, and instructions in use of assistive technology devices/adaptive equipment) direct one-on-one contact, each 15 minutes |
|  | 97542 | Wheelchair management (eg, assessment, fitting, training), each 15 minutes |
|  | 97597 | Removal of devitalized tissue from wound(s), selective debridement, without anesthesia (eg, high pressure waterjet with/without suction, sharp selective debridement with scissors, scalpel and forceps), with or without topical application(s), wound assessment, and instruction(s) for ongoing care, may include use of a whirlpool, per session; total wound(s) surface area less than or equal to 20 square centimeters |
|  | 97598 | total wound(s) surface area greater than 20 square centimeters |
|  | 97760 | Orthotic(s) management and training (including assessment and fitting when not otherwise reported), upper extremity(ies), lower extremity(ies) and/or trunk, initial orthotic(s) encounter, each 15 minutes |
|  | 97761, 97762 | Prosthetic(s) training, upper and/or lower extremity(ies), initial prosthetic(s) encounter, each 15 minutes |
|  | 97763 | Orthotic(s)/prosthetic(s) management and/or training, upper extremity(ies), lower extremity(ies), and/or trunk, subsequent orthotic(s)/prosthetic(s) encounter, each 15 minutes |
|  |  | **Other Procedures** |
|  | 93797 | Physician services for outpatient cardiac rehabilitation; without continuous ECG monitoring (per session) |
|  | 93798 | with continuous ECG monitoring (per session) |
|  | 94667 | Manipulation chest wall, such as cupping, percussing, and vibration to facilitate lung function; initial demonstration and/or evaluation |
|  | 94668 | subsequent |
|  | 97750 | FCE/Performance Test |
| Spinal surgery | 00630 | Anesthesia for procedures in lumbar region; not otherwise specified |
|  | 00670 | Anesthesia for extensive spine and spinal cord procedures |
|  | 22102,22103 | Partial excision of posterior vertebral component for intrinsic bony lesion, single vertebral segment; lumbar/additional segment |
|  | 22224, 22226 | Osteotomy of spine, including discectomy, anterior approach, single vertebral segment; lumbar/additional segment |
|  | 22558 | Arthrodesis, anterior interbody technique, including minimal discectomy to prepare interspace (other than for decompression); lumbar |
|  | 22585 | Arthrodesis, anterior interbody technique, including minimal discectomy to prepare interspace (other than for decompression); each additional interspace |
|  | 22612 | Arthrodesis, posterior or posterolateral technique, single level lumbar |
|  | 22630 | Arthrodesis, posterior interbody technique, single interspace; lumbar |
|  | 22802 | Arthrodesis, posterior, for spinal deformity, with or without cash 7–12 vertebral segments |
|  | 22840 | Insertion of spine fixation device |
|  | 22842–22844 | Posterior segmental instrumentation leg, pedicle fixation, dual rods with multiple hooks and sublaminal wires); 3–6 vertebral segments through 13 or more segments |
|  | 22851 | Apply spine prosthetic device |
|  | 20930 | Allograft for spine surgery only; morselized |
|  | 20936 | Spinal bone autograft |
|  | 63005 | Laminectomy with exploration and/or decompression of spinal cord and/or cauda equina, without facetotomy, foraminotomy, or discectomy, 1 or 2 vertebral segments; lumbar, except for spondylolisthesis |
|  | 63011,63012 | Laminectomy with exploration and/or decompression of spinal cord and/ar cauda equina, without facetectomy, foraminotomy, or discectomy, 1 or 2 vertebral segments; sacral or lumbar |
|  | 63030 | Laminotomy (hemilaminectomy), with decompression of nerve root(s), including partial facetectomy, foraminotomy, and/or excision of herniated intervertebral disk; 1 interspace, lumbar |
|  | 63035 | Laminotomy (hemilaminectomy), with decompression of nerve root(s), including partial facetectomy, foraminotomy, and/or excision of herniated intervertebral disk; each additional interspace, cervical or lumbar |
|  | 63042 | Laminotomy (hemilaminectomy), with decompression of nerve roars), including partial facetectomy, foraminotomy, and/or excision of herniated intervertebral disk, reexploration; lumbar |
|  | 63047 | Laminectomy, facetectomy, and foraminotomy (unilateral or bilateral, with decompression of spinal cord, cauda equina, and/or nerve root), single vertebral segment lumbar |
|  | 63048 | Removal of spine lamina−add-on |
|  | 63088–63091 | Vertebral corpectomy, partial or complete, combined thoracolumbar approach with decompression of spinal cord, cauda equina, or nerve root(s), lower thoracic or lumbar; each additional segment |
|  | 63185, 63190 | Laminectomy with rhizotomy |
|  | 62287 | Aspiration procedure percutaneous, of nucleus pulposus of intervertebral disk, any method, single or multiple levels; lumbar |
|  | 63200 | Laminectomy, with release of tethered spinal cord; lumbar |
|  | 63267,63272 | Laminectomy for excision or evacuation of intraspinal lesion other than neoplasm, extradural or intradural lumbar |
|  | 63290 | Laminectomy for biopsy/excision of intraspinal neoplasm; combined extraduratintradural lesion, any level |
|  | 63303 | Vertebral corpectomy, partial or complete, for excision of intraspinal lesion, single segment extradural, lumbar or sacral, by transperitoneal or retroperitoneal approach |
|  | 64622,64623 | Destruction by neurolytic agent; paravertebral facet joint nerve, lumbar, single level/additional level |
| Spinal Injection | 62311 | Injection, single, with or without contrast of diagnostic or therapeutic substance(s), epidural or subarachnoid; lumbar, sacral (caudal) |
|  | 64475, 64476 | Injection, paravertebral, lumbar/sacral |
|  | 64483-4 | Injection, anesthetic agent and/or steroid, transforaminal epidural; lumbar or sacral, single and additional levels |
|  | 72275 | Epidurography, radiological supervision and interpretation |
|  | 77003 | Fluoroscopic guidance and localization of needle or catheter tip for spine or paraspinous diagnostic or therapeutic injection procedures |
