# Supplementary material for: Initial treatment approaches and healthcare utilization among veterans with low back pain: a propensity score analysis
Source: BMC Health Serv Res. 2023 Mar 21;23:275. doi: 10.1186/s12913-023-09207-y (PMC10029316; doi:10.1186/s12913-023-09207-y)
Supplement: Supplementary file 4 — Supplementary Material 4 [file 12913_2023_9207_MOESM4_ESM.docx]

**Additional file 4**

| **Table A4: ICD Codes Identifying Mental Health and Substance Abuse Disorders** | | | |
| --- | --- | --- | --- |
| **ICD-9-CM/10 Code** | **Description** | **ICD-9-CM/10 Code** | **Description** |
| 296.xx/F30.10 | Major depressive disorder/Manic depression | 309.81/F43.1 | Post-traumatic stress disorder |
| 298.xx/F40.298 | Anxiety disorder | 291.xx/F10.99 | Alcohol use disorder |
| 300.xx/F41.9 | Anxiety disorder | 303.xx-305.xx  F10.229, F11.20, F10.10, F17.200, F12.10 | Alcohol, Opioid, and other drug dependency |
| 301.xx/F60.9 | Personality disorder | 648.3 | Drug dependency |
| 308.xx/F43.0/R45.7 | Unspecified acute reaction to stress | 309.xx/F43.20 | Adjustment disorder |
