# Supplementary material for: Initial treatment approaches and healthcare utilization among veterans with low back pain: a propensity score analysis
Source: BMC Health Serv Res. 2023 Mar 21;23:275. doi: 10.1186/s12913-023-09207-y (PMC10029316; doi:10.1186/s12913-023-09207-y)
Supplement: Supplementary file 5 — Supplementary Material 5 [file 12913_2023_9207_MOESM5_ESM.docx]

**Additional Material Legends**

Additional file 1, (.doc), Table A1: ICD Codes Used to Identify Low Back Pain

Table A1 lists all ICD-9-CM and ICD10 diagnostic codes used to identify low back pain as inclusion criteria for this study.

Additional file 2, (.doc), Table A2: ICD Codes Identifying Conditions Used as Exclusion Criteria

Table A2 lists all ICD-9-CM and ICD10 diagnostic codes used to identify excluded diagnoses.

Additional file 3, (.doc), Table A3: CPT-4 Codes Identifying Procedures

Table A3 lists all CPT-4 codes used to identify the following procedures: physical therapy, spinal surgery and spinal injection.

Additional file 4, (.doc), Table A4: ICD Codes Identifying Mental Health and Substance Abuse Disorders

Table A4 lists ICD-9-CM and ICD10 diagnostic codes used to identify mental health and substance abuse disorders relevant to this study.
